# The Role of Deubiquitinases in Virus Replication and Host Innate Immune Response

**DOI:** 10.3389/fmicb.2022.839624

**Published:** 2022-02-24

**Authors:** Qinglin Zhang, Qizhen Jia, Wenying Gao, Wenyan Zhang

**Affiliations:** ^1^College of Life Sciences of Jilin University, Changchun, China; ^2^Center for Pathogen Biology and Infectious Diseases, Institute of Virology and AIDS Research, Key Laboratory of Organ Regeneration and Transplantation of the Ministry of Education, The First Hospital of Jilin University, Changchun, China

**Keywords:** DUBs, ubiquitin, PTM, viral infection, antiviral innate immunity

## Abstract

As a critical post-translational modification, ubiquitination is known to affect almost all the cellular processes including immunity, signaling pathways, cell death, cancer development, and viral infection by controlling protein stability. Deubiquitinases (DUBs) cleave ubiquitin from proteins and reverse the process of ubiquitination. Thus, DUBs play an important role in the deubiquitination process and serve as therapeutic targets for various diseases. DUBs are found in eukaryotes, bacteria, and viruses and influence various biological processes. Here, we summarize recent findings on the function of DUBs in modulating viral infection, the mechanism by which viral DUBs regulate host innate immune response, and highlight those DUBs that have recently been discovered as antiviral therapeutic targets.

## Introduction

Post-translational modifications (PTMs) play an important role in altering proteins without changing the nucleotide sequence of the organism’s DNA or RNA, thus enhancing their response and adaptation to complex environmental variations. PTMS includes methylation, acetylation, phosphorylation, hydroxylation, and ubiquitination (Deribe et al., 2010). Among them, ubiquitination and deubiquitination significantly regulate various cellular processes in eukaryotes, such as signals transduction, protein trafficking, cell motility, transcription, apoptosis, cancer development (Yang et al., 2013; Seo et al., 2018; Tang et al., 2018; Mennerich et al., 2019; Jin et al., 2020; Li et al., 2021). Moreover, the ubiquitination and deubiquitination also exert diverse functions in archaea, bacteria, and viruses (Fuchs et al., 2018; Hermanns and Hofmann, 2019; Wu et al., 2020). The process of ubiquitination involves the conjugation of ubiquitin (Ub) or Ub-like modifiers (Ubl) [for example, the small ubiquitin-like modifier (SUMO), the ubiquitin-like protein (NEDD8), and IFN-induced 15-kd protein (ISG15)] to substrate proteins by three types of enzymes, including E1 ubiquitin-activating enzyme, E2 ubiquitin-conjugating enzyme, an E3 ubiquitin-protein ligase, and the sequential cascades of E1-E2-E3 enzymes have been well studied (Deribe et al., 2010; Zheng and Shabek, 2017; Hu et al., 2020). The important molecule ubiquitin contains seven lysine residues (K6, K11, K27, K29, K33, K48, and K63) and an N-terminus methionine (M1) serving as ubiquitination sites, which result in various polyubiquitin chain linkage types. “Ubiquitin code” is comprised by the architecture of polyubiquitin chain linkage-type, the number of modified sites, the length of the added ubiquitin molecules, and dictates the fate of the substrates (Komander and Rape, 2012). For example, the K48-linked polyubiquitin chain often leads to proteasome degradation of the protein substrate, whereas K63-linked polyubiquitin mainly involves non-degradative roles such as innate immunity or intracellular trafficking of modified proteins (Oshiumi et al., 2013; Song et al., 2016; Okamoto et al., 2017). The conjugation of ubiquitin is reversible since deubiquitinases (DUBs) can cleave peptide or isopeptide bonds between ubiquitin and substrate protein or conjoined ubiquitin molecules. “Ubiquitin code” also dictates a wide variety of distinct DUBs activities and preferences, which has been well-reviewed (Clague et al., 2019).

The study of molecular pathways of deubiquitination was initiated in the 1990s and then further investigated and elucidated (Tobias and Varshavsky, 1991; Baker et al., 1992; Papa and Hochstrasser, 1993; Wilkinson, 1997). DUBs are key effectors of the removal of ubiquitin, which reverses the fate of the modified proteins as well as the following molecular and cellular functions of the proteins (Clague et al., 2013; Kategaya et al., 2017; Clague et al., 2019). DUBs have nearly 100 family members, which are classified into seven families, ubiquitin-specific proteases (USPs), ovarian tumor proteases (OTUs), ubiquitin carboxyl-terminal hydrolases (UCHs), Ataxin-3 like proteins (Josephins), MIU-containing new DUB family (MIUDY), zinc-finger ubiquitin protease 1 (ZUB/ZUFSP), and Jab/MPN domain-associated met-alloisopeptidases (JAMMs) (Hermanns and Hofmann, 2019). Each possesses unique cleavage specificity owing to its structural characteristics (Mevissen and Komander, 2017).

The protein levels of individual DUB and their intracellular localization are important for DUBs function in the cellular process. The estimated copy number of DUBs ranges from the low hundreds (limit of detection) to hundreds of thousands per cell. A systemic subcellular localization screen by GFP (Green fluorescent protein)-tagged DUBs in HeLa cells, combined with individual studies, showed that DUBs distribute in a variety of defined structures, such as the nucleolus (USP36 and USP39), plasma membrane (USP6 and JOSD1), microtubule [USP21 and CYLD (cylindromatosis)], Golgi (USP33V3 and USP32) (reviewed in Clague et al., 2019). DUBs are associated with almost all biological functions and diseases. For example, USP14, CYLD, and UCHL1 regulate cardiac hypertrophy by the different mechanism (Wang et al., 2015; Liu et al., 2016; Bi et al., 2020; Qi et al., 2020). Some DUBs regulate tumorigenesis and migration by their intrinsic oncogenic or tumor suppressor activities (UCHL1 and CYLD), or by controlling key epigenetic changes that affect cancer development (USP22), or by affecting the protein levels and/or activities of various cancer-related proteins (USP7 and USP28) (Sacco et al., 2010; Nicholson and Suresh Kumar, 2011; Wang and Dent, 2014; Harrigan et al., 2018; Xiao et al., 2019). In addition, DUBs have also been reported to function in interferon (IFN) antiviral signaling, dependent or independent of their deubiquitinase activity (Wang L. et al., 2013; Zhong et al., 2013; Fan et al., 2014; Pauli et al., 2014; Zhang et al., 2015; Gu et al., 2017; Qian et al., 2018; Liu et al., 2020).

Virus infection induces a host immune response, and leads to multiple serious diseases, including fever, chronic hepatitis, acquired immunodeficiency syndrome (AIDS), and cervical cancer (Tanuma et al., 2016; De Nola et al., 2019; Gallardo et al., 2019; Thomas, 2019). Furthermore, COVID-19 caused by SARS coronavirus 2 (SARS-CoV-2) has resulted in over 12,340,000 cases and 2,710,000 deaths worldwide until the end of March 2021 (Yamamoto et al., 2020). Therefore, investigations have focused on the molecular mechanisms that regulate or mediate virus replication (García et al., 2017; Kumar et al., 2018; Luo et al., 2018; Zhu et al., 2020). DUBs are known to participate in virus-related cellular activities (Zhang et al., 2011; Saxena and Kumar, 2014). Isaacson and Ploegh (2009) had summarized the effect of Ub, Ubl, and deubiquitination in viral infection, and then Gu and Shi (2016) reviewed the mechanism used by viruses to bypass or employ DUBs to evade the host immune defense. As an interesting therapeutic target, the clinical development of selective DUB inhibitors has been well-reviewed (Harrigan et al., 2018). Here, we mainly summarize the recent studies on the role of DUBs in viral replication, including host-encoded- and virus-encoded DUBs, and the influence of DUBs on the innate immunity of the host. Our review aims to provide a brief insight into the above fields and highlight the potential therapeutic targets of virus infection.

## Host-Encoded Deubiquitinases

### Host-Encoded Deubiquitinases Regulate Viral Replication by Affecting Innate Immune Response

**FIGURE 1:**
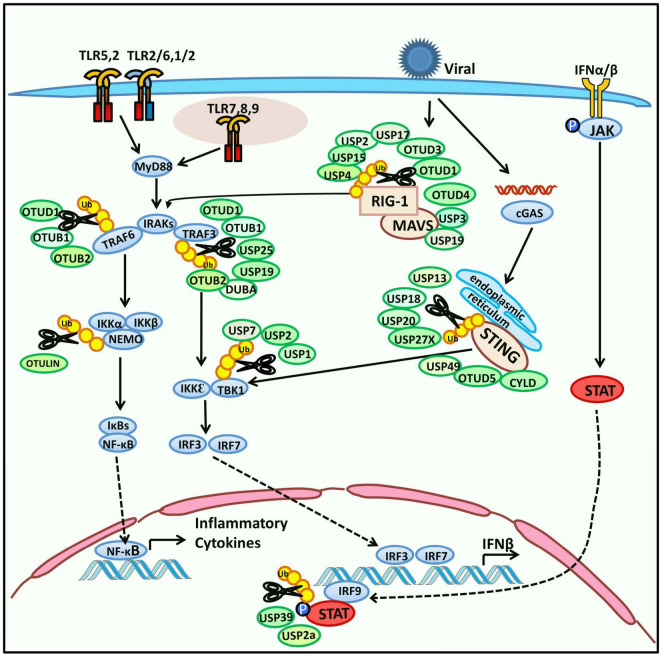
Regulation of host-encoded DUBs in innate immune response. Toll-like receptors (TLRs) are a type of pattern-recognition receptors (PRRs) that recognize pathogen-associated molecular patterns (PAMPs) and subsequently change their conformation to initiate downstream signaling. TLRs are located on the cell membrane, phagosome membrane, endosome, and endoplasmic reticulum (TLRs on endosomes and endoplasmic reticulum are not shown). TLRs can activate the expression of NF-κB or IRF3 and subsequently induce the expression of inflammatory cytokines or IFN-β in the nucleus. Activated TLRs recruit adaptor proteins, including TIRAP, MyD88, TRAM, and TRIF. First, the adaptors interact with IRAKs and TRAF6 and then activate NF-κB through the TAK1-IKK-IκB axis. Second, the adaptors mediate the expression of IRF3 *via* TRIF-TRAF3-TBK1 signaling. In addition, another PRR, RIG-I, which belongs to the RIG-I-like receptors (RLRs) family, is responsible for sensing RNA of pathogens. It interacts with MAVS, which further induces both NF-κB and IRF3. Stimulator of interferon genes (STING), located on the endoplasmic reticulum senses cytoplasmic DNA and regulates IFN signaling by activating TBK1 and IRF3. IFN-β induces the phosphorylation of JAK and expression of interferon-stimulated genes (ISGs) by activating the downstream JAK-STAT pathway. Host-encoded DUBs interact with the above signaling molecules to modulate the innate immune response. Their deubiquitinating function and substrates are shown in the schematic.

The innate immune response begins with pathogen recognition. Pathogen-associated molecular patterns (PAMPs) are structural characteristics that broadly exist in various pathogens and are recognized by germline-encoded pattern-recognition receptors (PRRs). Activated PRRs initiate cellular signaling pathways which defend against the invading microbes (Akira et al., 2006). PRRs include Toll-like receptors (TLRs), RIG-I-like receptors (RLRs), NOD-like receptors (NLRs), and C-type lectin receptors (CLRs). Both TLRs and RLRs are responsible for sensing viral infection. Upon recognition, TLRs recruit adaptor proteins, including MyD88 (Myeloid differentiation primary response protein 88), TIRAP (Toll/interleukin 1 receptor domain-containing adaptor protein), TRIF (toll/interleukin 1 receptor-domain-containing adapter-inducing interferon-β), and TRAM (a transverse rectus abdominis), and lead to activation of the TRAF3-TBK1-IRF3 axis to induce type I IFN (IFN-I), or the TRAF6-TAK1-IKK axis to activate nuclear factor κ-light-chain-enhancer of activated B cells (NF-κB) and stimulate inflammatory cytokines (Kawai and Akira, 2011; Lim and Staudt, 2013; Ivashkiv and Donlin, 2014). As a typical member of the RLRs family, Retinoic acid-inducible gene I (RIG-I) interacts with a mitochondrial activator of virus signaling (MAVS), the essential signaling adaptor protein of RIG-I, and causes the activation of TBK1 (TANK-binding kinase 1) and IKKε (IκB kinase ε) (Kell and Gale, 2015; Chow et al., 2018). As the TLR and RIG-I signaling have crosslinks and have been well characterized, we summarize recent studies on regulating host-encoded DUBs in viral replication *via* TLR, RIG-I, or STING signaling (Figure 1). In most cases, host DUBs exert their deubiquitinating activity to reverse the ubiquitination and degradation of key molecular regulators of TLR or RIG-I signaling, including TRAF3 (TNF-receptor-associated factor 3), TBK1, MAVS (Mitochondria antiviral signaling protein), and other molecules, resulting in their stabilization, thereby altering the strength of the innate immune response to restrict the proliferation of the virus. Located on the endoplasmic reticulum, STING (the stimulator of IFN genes) is responsible for sensing cytoplasmic DNA and exerts its function by activating TBK1 and IRF3 (IFN regulatory factor 3) thus stimulating IFN-I production (Tanaka and Chen, 2012).

Studies on host DUBs’ function in innate immune response could be dated to the beginning of the 21st century. The tumor suppressor CYLD was originally reported to be a deubiquitinase that negatively regulates NF-κB *via* its deubiquitinase activity in 2003 (Kovalenko et al., 2003; Trompouki et al., 2003). The subsequent studies showed that CYLD is a negative regulator targeting RIG-I by the removal of K63-linked RIG-I polyubiquitin chains, resulting in the inactivation of IKKε/TBK1, and eventually inhibiting IFN promoter (Friedman et al., 2008; Zhang et al., 2008). In 2018, CYLD was reported to positively regulate STING-mediated antiviral signaling by the cleavage of K48-linked ubiquitination of STING and the blockage of its degradation, thus sustaining the level of STING and triggering the antiviral response (Zhang et al., 2018b). Zinc finger protein A20 (A20) is an OTU domain DUB and also has E3 ubiquitin ligase activity. It has been reported that A20 functions as a potent inhibitor of NF-κB signaling through the cooperative activity of its two ubiquitin-editing domains or antagonizing the interactions between TRAF6, TRAF2, and cIAP1 with the E2 ligases Ubc13 and UbcH5c, which are required for TRAF6 ubiquitination and NF-κB activation (Wertz et al., 2004; Shembade et al., 2010). A20 has been reported to be a negative regulator of antiviral signaling by binding to TRIF and inhibition of TRIF-mediated activation of ISRE and IFN-β as well as Sendai virus (SeV) triggered NF-κB promoter, and blocks RIG-I-induced activation of NF-κ B-, IRF3- and IRF7-dependent promoters (Wang et al., 2004; Lin et al., 2006). So far, there are few studies of host-encoded DUBs other than USP and OTU family which regulate innate immune responses, so here we are focusing on these two DUB family members.

### Ubiquitin-Specific Proteases

Among the seven families of DUB families, the USP family is the largest DUB family, with more than 60 USPs identified (Yuan T. et al., 2018). Many USPs participate in the regulation of viral replication and are widely involved in RIG-I-induced antiviral immunity, such as USP17, USP3, USP4. In 2010, USP17 was identified in virus-induced IFN signaling, whose role in viral immunology is explored much earlier. Knockdown of USP17 increases the ubiquitination level of RIG-I and another RLR, MDA5, and reduces the production of SeV-induced IRF3 and IFN-β (Chen et al., 2010). It has been demonstrated that RIG-I K63-linked ubiquitination mediated by the E3 ubiquitin ligase tripartite motif protein 25 (TRIM25) is required for its activation and the stimulation of IFN signaling (Gack et al., 2007). In 2013, USP3 was reported to inhibit the type-I IFN pathway by binding to the K63-linked polyubiquitin chains of RIG-I and cleaving them, resulting in the negative regulation of RIG-I ubiquitination. Knockdown of USP3 promotes the phosphorylation of IRF3 and enhances the resistance of cells to viral infection (Cui et al., 2014). As a positive regulator of RIG-I-induced IFN signaling, USP4 interacts and removes K48-linked polyubiquitin chains of RIG-I and prevents RIG-I from degradation. Overexpression of USP4 suppresses VSV replication, while the knockdown of USP4 has the opposite effect (Wang L. et al., 2013). There are two contradictory reports about the regulation mechanism of USP15 on antiviral signaling (Pauli et al., 2014; Zhang et al., 2015). The first research reveals that USP15 deubiquitinates K48-linked ubiquitination of TRIM25 and prevents the degradation of TRIM25, which indicates that USP15 indirectly enhances TRIM25- and RIG-I-dependent production of IFN-I and the antiviral activity (Pauli et al., 2014). However, the other research claims that USP15 serves as a negative regulator of RIG-I signaling by deconjugating K63-linked polyubiquitin chains from RIG-I, resulting in the suppression of RIG-I-mediated antiviral responses. Interestingly, this research also points out that a part of this inhibition effect is independent of the DUB activity of USP15 (Zhang et al., 2015). Enterovirus 71 (EV71) infection induces the expression of host DUB USP19, and USP19 removes K63-linked ubiquitination of TRAF3, thereby inhibiting the antiviral IFN signaling. Knockdown of USP19 promotes cellular antiviral immunity and represses the replication of EV-71 (Gu et al., 2017). USP21 is the same negative regulator of the IFN signaling as USP19 by targeting and deubiquitinating K63-linked ubiquitination of RIG-I, though the specific site of deubiquitination has not been clarified (Fan et al., 2014). USP25 has been identified as a regulator of TLR signaling by removing the cIAP2 (cellular inhibitor of apoptosis 2)-mediated K48-linked ubiquitination of TRAF3, resulting in the blockage of TRAF3 degradation. The deficiency of USP25 enhances the extent of ubiquitination of TRAF3 and accelerates its degradation after TLR4 activation, which potentiates TLR4-induced activation of MAPK and NF-κB signaling, but suppresses the activation of IRF3 (IFN regulatory factor 3). Therefore, USP25 enables a balance between proinflammatory cytokines and type I IFNs by maintaining cellular TRAF3 level (Zhong et al., 2013).

USP1 forms a complex with USP1-associated factor 1 (UAF1), which reduces K48-linked ubiquitination of TBK1 and stabilizes it, resulting in the downstream activation of IRF3 and promotion of IFN-β secretion. The USP1-UAF1 inhibitor ML323 suppresses the expression of IFN-β and enhances virus replication *in vitro* and *in vivo*. This finding indicates a positive-regulatory effect of USP1 on IFN signaling (Yu et al., 2017). Another study reported that in contrast to USP1 and USP39, USP7 acts as a negative regulator of IFN signaling. Overexpression of USP7 enhances the stabilization of the E3 ligase TRIM27, thus promoting the ubiquitination and degradation of TBK1 and vesicular stomatitis virus (VSV) replication, whereas knockdown and knockout of endogenous USP7 inhibit TBK1 ubiquitination and degradation and increase IFN-β expression (Cai et al., 2018). Like USP7, USP5 is a restrictive factor for red-spotted grouper nervous necrosis virus (RGNNV)-induced IFN response in sea perch. Overexpression of USP5 leads to inhibition of other RLR signaling pathway-related genes, including MAVS, TRAF3, and TBK1, and strongly represses the IFN-I promoter’s activation in zebra fish increases RGNNV replication *in vitro*. However, the mechanism by which USP5 influences RLR signaling genes needs to be elucidated further (Jia et al., 2020). Knockdown of USP39, another positive regulator of the IFN pathway, notably promotes viral replication. Interestingly, USP39 does not influence IFN-I production but affects IFN downstream JAK/STAT signaling. USP39 downregulates STAT K6-linked ubiquitination, thus stabilizing STAT (Peng et al., 2020). USP2a also targets STAT and functions as a regulator of antiviral signaling. STAT is mainly located in the nucleus, and USP2a could translocate into the nucleus and reduce the K48-linked ubiquitination and degradation of pY701-STAT1 (STAT activation depends on phosphorylation at tyrosine 701). Therefore, USP2a maintains the level of pY701-STAT induced by IFN and promotes antiviral immunity (Ren et al., 2016). USP2b targets TBK1 and negatively regulates IFN-β signaling by the cleavage of K63-linked polyubiquitin chains of TBK1, thus inhibiting the kinase activity of TBK1. Overexpression of USP2b promotes the replication of vesicular stomatitis virus (VSV), whereas the knockdown of USP2b represses VSV replication (Zhang et al., 2014).

As a negative regulator of cellular antiviral responses that targets STING, USP13 deconjugates K27- and K63-linked polyubiquitin chains of STING and then prevents the recruitment of TBK1 to STING. The knockdown of USP13 activates IRF3 and NF-κB and enhances antiviral immunity. Consistently, the deficiency of USP13 inhibits Herpes simplex virus (HSV-1) infection (Sun et al., 2017). USP49 has a similar function as USP13 (Ye et al., 2019). USP18 recruits USP20 and deconjugates K48-linked ubiquitination of STING, thereby protecting STING from proteasomal degradation and promoting the production of type-I IFN. The knockdown of USP20 or USP18 promotes STING ubiquitination and degradation and impairs IRF3 and NF-κB pathways, thereby reducing the resistance of mice to HSV-1 infection (Zhang et al., 2016). USP27X also targets, and the mechanism involves cyclic GMP-AMP synthase (cGAS), a cytosolic DNA sensor catalyzing the formation of a second messenger cGAMP. cGAMP binds to STING and promotes the IFN-I signaling. USP27X interacts with cGAS and removes its K48-linked ubiquitination, thereby leading to the stabilization of cGAS and the enhancement of IFN production (Guo et al., 2019).

### Ovarian Tumor Proteases

Deubiquitinases in the OTU superfamily were originally identified from different organisms and affect antiviral immunity as well as virus proliferation (Kayagaki et al., 2007; Li et al., 2010). Deubiquitinating enzyme A (DUBA) was identified in 2007 to act as a negative regulator of IFN signaling, which removes K63-linked ubiquitination of TRAF3, resulting in the dissociation of TRAF3 from TBK1 (Kayagaki et al., 2007). OTUB1 and OTUB2 negatively regulate type-I IFN signaling by deubiquitinating TRAF3 and TRAF6 after viral infection (Li et al., 2010). OTUD1 could also inhibit IFN response. OTUD1 could be induced by an RNA virus and could upregulate the level of protein Smurf1. Smurf1 could ubiquitinate and degrade each component of the MAVS/TRAF3/TRAF6 signalosome (Zhang et al., 2018a). OTUD3 is an acetylation-dependent DUB. Its DUB activity relies on K129 acetylation, and it could remove the K63-linked ubiquitination of MAVS, hence repressing antiviral response. OTUD3 acetylation would be removed upon virus infection, and the DUB activity would be blocked. Therefore, OTUD3 is controlled to timely response to virus infection (Zhang et al., 2020). OTUD5 exerts DUB activity by cleaving STING K48, enhancing STING stability, and thus promoting type-I IFN signaling (Guo et al., 2020). Similarly, OTUD4 deubiquitinates and stabilizes MAVS and promotes antiviral signaling. Knockdown of OTUD4 inhibits the activation of NF-κB and IRF3, resulting in the suppression of their downstream genes, thus enabling the VSV replication (Liuyu et al., 2019). OTULIN is an ovarian tumor domain deubiquitinase that possesses linear linkage specificity. PRRSV infection enhances the expression of OTULIN, and overexpression of OTULIN increases PRRSV propagation. OTULIN is recruited by PRRSV Nsp11 and removes linear ubiquitination targeting NEMO, a subunit of the IKK complex, resulting in the reduction of IFN-I production (Su et al., 2018).

### Host-Encoded Deubiquitinases Modulate Virus Replication *via* Viral Proteins

In addition to modulating innate immune signaling pathways, host DUBs also regulate viral replication *via* the interaction with virus-encoded proteins. Due to the selective pressure of evolution, viruses have evolved to recruit host proteins to assist their propagation, and host deubiquitinases are widely involved (Table 1). USP7 is the first identified DUB that interacts with ICP0 of HSV-1, mediating the stability of ICP0 or modulating TLR-mediated innate response (Everett et al., 1997; Boutell et al., 2005; Daubeuf et al., 2009). USP7 has been demonstrated to participate in the replication of multiple viruses, including Epstein-Barr virus (EBV), Merkel cell polyomavirus (MCPyV), and Human immunodeficiency virus type 1 (HIV-1) (Holowaty and Frappier, 2004). USP7 directly binds to the large T (LT)-antigen of MCPyV, an early viral gene product that plays a significant role in viral replication, which promotes the binding affinity of LT to the viral origin of replication and decreases viral DNA replication. This process is not related to the deubiquitinase activity of USP7 (Czech-Sioli et al., 2020). USP7 also serves as an enhancer of viral replication. Inhibition of USP7 decreases the replication of human adenoviral species C (HAdV-C5), most adenoviral species A as well as clinical isolates, and HIV (Ali et al., 2017; Kosulin et al., 2018). HIV Tat is produced in the early phase of HIV infection and is mainly responsible for transacting transcription and promoting viral production. USP7 stabilizes Tat by deubiquitylating and blocking it from proteasomal degradation, resulting in the promotion of virus propagation (Ali et al., 2017). In EBV infected cells, the EBNA1 protein binds to specific recognition sites *oriP* in the latent origin of replication required for the viral genome’s replication and maintenance. USP7 greatly stimulates EBNA1-DNA interactions and enhances the ability of EBNA1 to activate transcription by modifying histone in a deubiquitinase-dependent manner (Sarkari et al., 2009). Similarly, in Kaposi’s sarcoma herpesvirus (KSHV), USP7 interacts with the viral latency-associated nuclear antigen 1 (LANA), which is involved in latent viral replication and maintenance of the viral genome, thus enhancing the latent viral DNA replication of KSHV (Holowaty et al., 2003; Jager et al., 2012). vIRF1 of KSHV was identified as a novel interaction partner of USP7, which deregulates USP7 to inhibit p53-mediated antiviral responses (Chavoshi et al., 2016).

**TABLE 1:** Host-encoded DUBs and the targeted viruses, proteins, or genes.

Host-encoded DUBs	Targeted viruses or genes, proteins	The mechanism	References
USP1/USP12/USP46	Human papillomavirus (HPV) E1 helicase	Unwinding DNA and enhancing viral DNA replication	Lehoux et al., 2014
USP14	Murine norovirus 1 (MNV-1) VPg gene	Non-structural gene	Perry et al., 2012
USP 7	HIV-1 Tat	Promoting viral production	Ali et al., 2017
	Herpes simplex virus type 1 (HSV-1) ICP0 (Vmw110)	Non-specific activator of gene expression	Everett et al., 1997
	Epstein-Barr virus (EBV) EBNA1	Initiating EBV latent genome replication	Sarkari et al., 2009
	Kaposi’s sarcoma herpesvirus (KSHV) LANA vIRF1	Working in latent viral replication Inhibit p53-mediated antiviral responses	Chavoshi et al., 2016
	Merkel cell polyomavirus (MCPyV)	Working in viral replication	Czech-Sioli et al., 2020
	red-spotted grouper nervous necrosis virus (RGNNV)	Strongly represses the activation of the IFN-I promoter	Jia et al., 2020
	TRIM27	Enhances the stabilization of the E3 ligase TRIM27	Cai et al., 2018
USP15	HIV Nef	Necessary for AIDS pathogenicity	Pyeon et al., 2016
	HIV Gag	HIV structural protein	
USP11	Influenza A virus RNA	A component of viral RNA replication complex	Liao et al., 2010
OTULIN	PRRSV Nsp11	OTULIN is recruited by PRRSV Nsp11 and removes linear ubiquitination targeting NEMO	Su et al., 2018
USP19, DUBA	TRAF3	Removes K63-linked ubiquitination of TRAF3	Kayagaki et al., 2007; Gu et al., 2017
USP25	TRAF3	The enhancement of TRAF3 degradation	Zhong et al., 2013
CYLD; USP18 and USP20	STING	The cleavage of K48-linked ubiquitination of STING	Zhang et al., 2016; Zhang et al., 2018b
USP13/49	STING	Deconjugates K27- and K63-linked polyubiquitin chains of STING	Sun et al., 2017; Ye et al., 2019
USP17	RIG-I	Increases the ubiquitination level of RIG-I	Chen et al., 2010
USP3/21/19	RIG-I	Removes K63-linked polyubiquitin chains of RIG-I	Cui et al., 2014; Fan et al., 2014
USP4	RIG-I	Interacts and removes K48-linked polyubiquitin chains of RIG-I	Wang L. et al., 2013
USP15	TRIM25/RIG-I	Enhanced the TRIM25- and RIG-I-Dependent production of IFN-I and suppressed RNA virus replication	Pauli et al., 2014; Zhang et al., 2015
USP1	TBK1	Reduces K48-linked ubiquitination of TBK1 and stabilizes it	Yu et al., 2017
USP2a	STAT	Reduce the K48-linked ubiquitination and degradation of pY701-STAT1	Ren et al., 2016
USP39	STAT	Downregulates STAT K6-linked ubiquitination, thus stabilizing STAT	Peng et al., 2020
USP2b	TBK1	Inhibits the kinase activity of TBK1	Zhang et al., 2014
USP27X	cGAS-STING	Removes cGAS K48-linked ubiquitination	Guo et al., 2019
DUBA	TRAF3	Removes K63-linked ubiquitination of TRAF3	Kayagaki et al., 2007
OTUB1, OTUB2	TRAF3 and TRAF6	Deubiquitinating TRAF3 and TRAF6	Li et al., 2010
OTUD1	Smurf1	Upregulate the level of protein Smurf1	Zhang et al., 2018a
OTUD3, OTUD4	MAVS	Deubiquitinates and stabilizes MAVS	Liuyu et al., 2019; Zhang et al., 2020
OTUD5	STING	Cleaving STING K48, enhancing STING stability	Guo et al., 2020

In high-risk human papillomavirus (HPV)-induced cancer, USP46 recruited by oncoprotein E6 leads to deubiquitination and stabilization of Cdt2/DTL, a component of the E3 ligase CRL4^Cdt2^, and represses the level of epigenetic regulator Set8, which promotes cell proliferation. Thus, depletion of USP46 inhibits HPV-transformation-induced tumor growth through the E6-USP46-Cdt2-Set8 pathway (Kiran et al., 2018). HPV E1 helicase, which possesses DNA unwinding activity and enhances viral DNA replication, could form a complex with UAF1 and any one of USP1, USP12 and USP46. Besides USP1, the catalytic activity-negative USP12 and USP46 also decrease viral replication, and E1-UAF1 binding is essential for the effect (Lehoux et al., 2014). Therefore, USP1, USP12, and USP46 promote viral replication *via* interacting with the E1-UAF1 complex.

Similarly, inhibition of USP14 causes the repression of replication of several flaviviruses, indicating the crucial role of USP14 in viral propagation (Nag and Finley, 2012; Perry et al., 2012). WP1130, a small molecule inhibitor, can inhibit Murine norovirus 1 (MNV-1) replication, and chemical proteomics showed that USP14 is the target of WP1130. The downregulation of USP14 by pharmacologic inhibition or siRNA-mediated knockdown reduces the expression of the MNV-1 non-structural gene VPg, suggesting the essential role of USP14 in effective MNV-1 inhibition (Perry et al., 2012).

Host DUBs also block the activity of crucial proteins during viral replication, thus exhibiting a negative regulatory function. Liao et al. reported that downregulation of USP11 results in higher level of influenza A virus (IAV) RNA, indicating that USP11 inhibits IAV replication. Moreover, the inhibitory effect of USP11 on viral genome replication requires its deubiquitinase activity. USP11 interacts with and deubiquitinates the nucleoprotein (NP) protein, a component of the viral RNA replication complex (Liao et al., 2010). In contrast to the positive role of USP7 in HIV-1 replication, USP15 represses the replication of HIV-1 by inducing the degradation of HIV-1 Nef and Gag proteins (Pyeon et al., 2016).

## Virus-Encoded Deubiquitinases

Deubiquitinases are widely presented in various viruses and significantly influence viral activity. During viral infection, a set of DUBs encoded by viruses mainly target various signaling molecules of innate immune pathways to suppress antiviral immunity, thus ensuring viral replication (Kumari and Kumar, 2018). In addition, some virus-encoded DUBs regulate viral replication through other mechanisms or mechanisms remaining unknown. Most viral DUB activity is mediated by papain-like proteases (PLPs) (Zheng et al., 2008; Frieman et al., 2009; Wang G. et al., 2011; Sun et al., 2012; van Kasteren et al., 2013; Xing et al., 2013; Chen et al., 2014; Yang et al., 2014; Yuan et al., 2015; Li et al., 2016). The adenovirus protease adenain was initially demonstrated to possess a deubiquitinating function in *in vitro* experiments (Balakirev et al., 2002). Then viral PLPs encoded by coronavirus family member such as SARS-CoV was reported to remove ubiquitin from substrate proteins (Barretto et al., 2005; Lindner et al., 2005; Bekes et al., 2016; Shin et al., 2020). UL36 encoded by HSV-1 and Marek’s disease virus (MDV), UL48 of HCMV are ubiquitin-specific cysteine proteases and have deubiquitinating activity (Kattenhorn et al., 2005; Wang et al., 2006; Jarosinski et al., 2007). DUB activity is strictly conserved in several other herpesviruses such as EBV, which encodes BPLF1 exhibiting deubiquitinating activity (Whitehurst et al., 2009). DUBs encoded by other viral genes are relatively few. For example, the DUB of nairoviruses resides in the polymerase protein but is not essential for RNA replication (van Kasteren et al., 2012). Previous studies have systematically summarized the role of viral DUBs in innate immunity (Gu and Shi, 2016; Zheng and Gao, 2020), here, we focus on the recent studies on how viral DUBs modulate the innate immune response and detail the signaling pathways that involve viral DUBs (Table 2).

**TABLE 2:** Virus-encoded DUBs and the targeted proteins or genes.

Virus-encoded DUBs	Targeted protein(s) or gene(s)	Mechanism	References
Adenovirus protease Adenain	N/A	Deubiquitinates a number of cellular proteins	Balakirev et al., 2002
SARS PLP	ISG15	Cleave replicase substrates; cleave K48-linked polyUb chains of ISG15	Barretto et al., 2005; Lindner et al., 2005; Bekes et al., 2016; Shin et al., 2020
	TRAF3, TRAF6	Inhibits the production of IFNs and pro-inflammatory cytokines	Li et al., 2016
HcoV-NL63 PLP2	N/A	Antagonizes the induction of IFN-I independent of protease and DUB activity	Clementz et al., 2010; Yuan et al., 2015
HSV-1 UL36	Proliferating cell nuclear antigen (PCNA)	Blocks the polymerase-η-mediated DNA damage-response translation synthesis (TLS) pathway	Kattenhorn et al., 2005
	TRAF3, ISRE promoter	Inhibits IFN-I production or counteracts the IFN-I-mediated signaling pathway	Wang S. et al., 2013; Yuan H. et al., 2018
	IκBα	The inactivation of the NF-κB pathway and the inhibition of IFN-β production	Ye et al., 2017
MDV UL36	N/A	Contributes to malignant outgrowths	Jarosinski et al., 2007
HCMV UL48	N/A	Facilitates production of infectious virus	Wang et al., 2006; Kim et al., 2009
	TRAF6, TRAF3, IRAK1, IRF7, STING	Promotes oncogenesis by inhibiting innate immunity of the host	Kumari et al., 2017
	RIP1	Inhibits TNFα-induced NF-κB activation	Kwon et al., 2017
EBV BPLF1	EBV ribonucleotide reductase (RR)	Interacts with, deubiquitinates, and influences the activity of the EBV RR	Whitehurst et al., 2009
	IκBα, TRAF6, NEMO	Suppresses TLR-mediated activation of NF-κB	Saito et al., 2013; van Gent et al., 2014
	PCNA	Attenuates polymerase η recruitment to DNA damage sites	Whitehurst et al., 2012
CCHFV OTU	NP	Associating with viral genomic RNA to form RNP complexes, initiate replication or transcription	Scholte et al., 2019
KSHV ORF64	RIG-I	Inhibition of RIG-I-mediated signaling	Inn et al., 2011
Arteri- and nairovirus DUBs	RIG-I	Control innate immune signaling	van Kasteren et al., 2012
TYMV PRO domain	66K RdRp	Ensures the replication of viral RNA	Fieulaine et al., 2020
HEV PCP	RIG-I, TBK1	Reduces IFN production	Nan et al., 2014
FMDV Ppro	RIG-1, TBK1, TRAF3, TRAF6	Blocks the IFN-β promoter	Wang D. et al., 2011
PEDV PLP2	IRF3	Negatively regulates type I interferon pathway	Xing et al., 2013
TGEV PL1	RIG-I, STING	Antagonizes production of IFN- β	Hu et al., 2017
PRRSV nsp2	IκBα	Antagonizes the type I interferon induction by interfering with the NF-kappaB signaling pathway	Sun et al., 2010
MHV-A59 PLP2	IRF3 TBK1	Blocks IRF3-, TBK1- and CARDIF-mediated IFN-β reporter activity	Zheng et al., 2008; Wang G. et al., 2011

*N/A represents not applicable.*

### Effect of Virus-Encoded Deubiquitinases on Retinoic Acid-Inducible Gene-I and Toll-Like Receptor Signaling

Viral DUB could influence the TLR pathway to antagonize innate immunity, and PLP2 encoded by Human coronavirus NL63 (HCoV-NL63) is a typical DUB targeting TLR signaling. HCoV-NL63 PLP2 was identified to possess DUB activity, and it could cleave both K48- and K63-linked polyubiquitin chains, thereby antagonizing IFN, although the inhibition effect is independent of its DUB activity (Clementz et al., 2010). In 2015, another study suggested one potential mechanism of HCoV-NL63 PLP2 antagonism to IFN signaling, but it is associated with PLP2 DUB activity. PLP2 is claimed to induce p53 degradation *via* deubiquitination and stabilization of the oncoprotein MDM2, an E3 ligase degrading p53. p53 could activate the transcription of IRF7. Hence, PLP2 blocks IFN-β signaling through the MDM2-p53-IRF7 pathway and facilitates viral replication (Yuan et al., 2015).

Multiple viral DUBs mainly target RIG-I signaling to regulate IFN response. The DUB activity of Crimean-Congo Hemorrhagic Fever virus (CCHFV) OTU is essential for inhibiting RIG-I-mediated IFN-β response and inactivated OTU DUB suppresses viral replication. OTU represses the RIG-I-mediated IFN-β response by removing ubiquitin (Ub) from RIG-I CARDs. Moreover, OTU DUB also inhibits the IFN-β response by blocking the activation of both IRF3 and NF-κB, indicating that CCHFV OTU DUB ensures the sufficient replication of the virus *via* multiple cellular signaling pathways (Scholte et al., 2017). KSHV ORF64 DUB, both arteri- and nairovirus DUBs decrease the ubiquitination of RIG-I, thus inactivating RIG-I-mediated IFN signaling (Inn et al., 2011; van Kasteren et al., 2012). DUBs can also repress MAVS-mediated IFN-β induction (van Kasteren et al., 2012). The interaction between herpesvirus BPLF1 and the 14-3-3-TRIM25 complex is essential for the induction of TRIM25 aggregates. TRIM25 ubiquitinates RIG-I, thus promoting RIG-I-triggered IFN signaling (Gupta et al., 2019).

Some viral DUBs can act on various RIG-I and TLR pathways adaptors. SARS-CoV infection inhibits IFN production as well as the upregulation of IFN-stimulated genes. SARS-CoV PLP interacts with IRF3 to block its phosphorylation and translocation into the nucleus and interrupts IRF3-mediated IFN signaling of either RIG-I or TLR pathway (Devaraj et al., 2007). However, a later study reported that SARS-CoV PLP does not directly interact with IRF3 and inhibit IRF3 phosphorylation *in vitro* but suppresses IRF3 signaling. PLP blocks both IRF3 and NF-κB signaling by deubiquitinating IKBα, while DUB activity of PLP is necessary for pathway antagonism but not sufficient by itself (Frieman et al., 2009). In addition, SARS coronavirus PLP also suppresses the TLR7 signaling pathway by removing K63-linked polyubiquitin of TRAF3 and TRAF6, thereby inhibiting the production of IFNs and pro-inflammatory cytokines (Li et al., 2016). Seneca Valley Virus (SVV) 3C protease represses the ubiquitination of RIG-I, TBK1, and TRAF3, thus inhibiting the expression of IFN-β and promoting virus replication (Xue et al., 2018). The PCP domain of hepatitis E virus (HEV) deubiquitinates RIG-I and TBK-1, thereby reducing IFN production (Nan et al., 2014). The leader proteinase (L^pro^) of the foot-and-mouth disease virus (FMDV) has DUB activity and strongly reduces ubiquitination of several key molecular regulators in the IFN-I response pathway, including RIG-1, TBK1, TRAF3, and TRAF6. Loss of DUB activity abolishes the ability of L^pro^ to block the IFN-β promoter (Wang D. et al., 2011).

### Effect of Virus-Encoded Deubiquitinases on STING Signaling

Some virus-encoded DUBs act on STING and influence downstream signaling. HSV-1 VP1-2 blocks IFN-I production by directly interacting with and deubiquitinating STING. In contrast, the VP1-2 mutant that lacks DUB activity enhances IFN expression due to increased STING ubiquitination as well as STING, TBK1, and IRF3 phosphorylation (Bodda et al., 2020). In addition, ORF64 DUB of MHV68 is essential for antagonizing the STING-dependent pathway which is induced by viral genomic DNA (Sun et al., 2015).

Human cytomegalovirus (HCMV) pUL48 blocks PRR-mediated IFN-I signaling through deubiquitinating a variety of key molecules, including STING, TRAF3, TRAF6, IRAK1, and IRF7 (Kumari et al., 2017). Upon ectopic expression, both CoV-NL63 PLP2 and SARS-CoV membrane-anchored PLpro domain (PLpro-TM) inhibit the formation of STING dimer and block the assembly of STING-MAVS-TBK1/IKKε complexes, thus inhibiting the activation of IRF3 and disrupting the signaling of IFN induction (Sun et al., 2012). It has also been demonstrated that SARS-Cov PLpro-TM represses the ubiquitination of RIG-I, STING, TRAF3, TBK1, and IRF3, thus inhibiting the assembly of the STING-TRAF3-TBK1 complex and the activation of IFN expression (Chen et al., 2014).

Some DUBs directly interact with RIG-I or STING and deubiquitinate them, resulting in suppression of STING- and RIG-I-mediated IFN-β signaling, such as porcine epidemic diarrhea virus (PEDV) PLP2 and transmissible gastroenteritis virus (TGEV) PL1 (Xing et al., 2013; Hu et al., 2017).

### Effect of Virus-Encoded Deubiquitinases on NF-κB Signaling

Some viral DUBs target NF-κB signaling to manipulate immune responses (Sun et al., 2010; Kwon et al., 2017; Ye et al., 2017). HSV-1 UL36 and porcine reproductive and respiratory syndrome virus (PRRSV) nsp2 both deubiquitinate K48-linked IκBα and prevent its degradation, resulting in the inactivation of the NF-κB pathway and the inhibition of IFN-β production (Sun et al., 2010; Ye et al., 2017). UL48 and UL45 encoded by HCMV that contain DUB activity, target receptor-interacting protein kinase 1 to inhibit NF-κB signaling (Kwon et al., 2017). EBV BPLF1 deubiquitinates IκBα, TRAF6 and NEMO, thus suppressing the activation of the NF-κB pathway at multiple steps. BPLF1 also inhibits NF-κB upstream TLR signaling, and reduces the production of IL-8 (van Gent et al., 2014). EBV BPLF1 deubiquitinates TRAF6, which is further associated with latent membrane protein 1 (LMP1) and inhibits NF-κB signaling, thereby increasing EBV DNA replication (Saito et al., 2013). SARS-CoV PLP antagonizes IFN and NF-κB signaling pathway by interfering with the activation of the important signaling proteins, IRF3 and IκBα in the respective pathways (Frieman et al., 2009).

### Effect of Virus-Encoded Deubiquitinases on Crosslinking Signaling Molecules

Besides the DUBs listed above, some viral DUBs act on the common downstream molecules of the TLR/RIG-I/STING signaling pathways. MHV-A59 PLP2 deubiquitinases IRF3 and inhibits its nuclear translocation *via* its DUB activity, thereby blocking IRF3-, TBK1-, and CARDIF-mediated IFN-β reporter activity (Zheng et al., 2008). Further studies also show that MHV-A59 PLP2 deubiquitinates TBK1 and inhibits its kinase activity, thereby preventing IRF3 from phosphorylation and inactivating IFN-β signaling (Wang G. et al., 2011). HSV-1 UL36USP deubiquitinates TRAF3 and prevents recruitment of TBK1, and inactivates the IFN-β promoter (Wang S. et al., 2013). It also binds to the IFNAR2 subunit, thus preventing JAK1-IFNAR2 interaction. Therefore, UL36USP antagonizes IFN-mediated JAKs and STAT activation (Yuan H. et al., 2018). MERS-Cov PLpro inhibits IRF3 phosphorylation and nuclear translocation, thus antagonizing the IFN-β pathway (Yang et al., 2014).

### Other Mechanisms of Virus-Encoded Deubiquitinases in the Regulation of Viral Replication

In several RNA viruses, virus-encoded DUBs affect and mediate viral replication. CCHFV OTU is required for viral replication, and occupancy of the synthetic ubiquitin variant (UbV-CC4) on CCHFV OTU directly interrupts viral RNA synthesis, thus blocking viral replication without targeting IFN signaling. CCHFV replication requires NP protein to associate with viral genomic RNA to form ribonucleoprotein (RNP) complexes and then interact with the L protein to initiate replication or transcription. In the presence of UbV-CC4, fewer NPs interact with the L protein, which indicates that UbV-CC4 inhibits CCHFV replication by interfering with the formation of replication complexes (Scholte et al., 2019). Turnip yellow mosaic virus (TYMV) PRO/DUB enzyme mediates the RNA-dependent RNA polymerase (RdRp) level, thereby regulating viral replication. The PRO domain cleaves TYMV-encoded 206 K polyprotein and produces 66 K RdRp. RdRp undergoes ubiquitination and degradation, whereas the DUB activity inhibits the degradation of the 66K RdRp and ensures the replication of viral RNA (Fieulaine et al., 2020). Among DNA viruses, HSV UL36USP and EBV BPLF1 enhance the sensitivity of cells to DNA-damaging agents to facilitate virus proliferation through its DUB activity (Whitehurst et al., 2012; Dong et al., 2017). In brief, UL36USP or BPLF1 deubiquitinates proliferating cell nuclear antigen (PCNA) and inhibits the subsequent formation of polymerase-η (pol-η) foci, thereby blocking the pol-η-mediated DNA damage-response translation synthesis (TLS) pathway (Dong et al., 2017).

Some DUB-mediated mechanisms have not yet been elucidated. PRRSV PLP2 possesses DUB activity and is essential for viral replication. However, the DUB activity is independent of the TNF-α pathway (Zhou et al., 2019). CCHFV L protein exhibits DUB activity but does not affect virus proliferation and innate immune responses (Tchesnokov et al., 2020). The reduction of FMDV DUB activity results in virus attenuation, but it is independent of interference with the expression of IFN (Medina et al., 2020). Murine cytomegalovirus (MCMV) encodes M48 DUB and MCK2 (MCMV-encoded chemokine 2), a viral C-C chemokine that enhances the host immune response. Blocking M48 DUB activity reduces MCMV replication, at least in the natural host, and promotes MCK2-induced immune response (Hilterbrand et al., 2017).

## Deubiquitinases Serve as Targets for Therapy

Being intrinsically attractive targets for drug development, the progress in the development of DUBs inhibitors or pharmacological modulation of DUB activities, in particular, the disease ranging from oncology to neurodegeneration, have been well-reviewed (Harrigan et al., 2018). As DUBs are crucial for virus replication, and participate in multiple signaling pathways during immune response, they present ideal targets for diagnosing and treating viral infections.

Perry et al. (2012) demonstrated that WP1130, a small molecule inhibitor of a subset of cellular DUBs, displays antiviral activity against murine and human norovirus, suggesting the importance of DUBs in modulating virus replication. Compound 9, a novel small molecule inhibitor derived from WP1130, markedly reduces the replication of murine norovirus, and amplifies the Norwalk virus genome (Charbonneau et al., 2014). A set of WP1130 derivatives retain the broad-spectrum antiviral function, acting on a variety of RNA viruses, including Sindbis virus and LaCrosse virus (Gonzalez-Hernandez et al., 2014). In addition, the 2-cyano-3-acrylamide compound C6 also facilitates the suppression of intracellular replication of murine norovirus by repressing DUB activity (Passalacqua et al., 2016). Overexpression of gga-miR-30d reduces the proliferation of infectious bronchitis virus (IBV), and USP47 is a target of gga-miR-30d in cells (Li et al., 2020). The DUBs inhibitors P22077 and PR-619 targeting USP7 and USP47 impair Gag processing, thereby blocking HIV-1 replication (Setz et al., 2017).

Efforts have also been focused on developing inhibitors against virus DUBs, and some have shown promise to be efficient antiviral drugs. The inhibitors targeting EBV DUB BPLF1, or thiopurine analog 6-mercaptopurine (6MP) and 6-thioguanine (6TG) targeting SARS-CoV PLpro all have been proved to be effective and promising new therapeutic strategies (Chen et al., 2009; Atkins et al., 2020). Thiopurine analog 6MP and 6TG that have long been used in cancer chemotherapy were found not only to be specific inhibitors for the SARS-CoV PLpro but also to be potential inhibitors of USP14 by computer docking analysis (Chen et al., 2009). GRL0617 binds to SARS-CoV PLP, abolishes its deubiquitinase activity, and thus inhibits viral replication (Ratia et al., 2008). HCMV UL48 and HSV UL36 execute DUB activity in K63 and K48 ubiquitin linkages. HCMV UL48 C24 mutant with mutations in the active site residues that completely abolish DUB activity results in a notable decrease in HCMV viral protein expression and virus proliferation (Kim et al., 2009). Furthermore, naphthalene inhibitor targeting SARS-CoV-2 PLP suppresses the activity of PLP and viral replication by interfering with its deubiquitinating activity (Ratia et al., 2006; Freitas et al., 2020).

## Conclusion and Perspective

Deubiquitinases are crucial regulators of viral replication and exert their function in multiple steps of signaling pathways to modulate host innate immune response. Among them, host-encoded DUBs play an indispensable role in PTMs, as they reverse the process of ubiquitination and allow for the fine-tuning of Ub modification. As key regulators of viral replication, host DUBs have two major functional mechanisms. First, they widely participate in innate immune pathways, deubiquitinate and stabilize critical signaling molecules, thus enhancing the strength of the antiviral immune response. Second, host DUBs interact with viral proteins that are important for replication and affect viral replication positively or negatively. In addition, viruses encode DUBs or protein domains that possess DUB activity.

In most cases, the biological function of viral DUBs is to antagonize the host immune system and ensure its proliferation. Like host DUBs, viral DUBs may also be involved in many steps of innate immune signaling to suppress their function. Moreover, viral DUBs regulate viral replication through a variety of mechanisms. Based on these biological roles, both host and viral DUBs represent potential targets for treating diseases.

For DUB-targeted therapy, more effort is needed to further elucidate the cellular pathways involving DUBs and identify DUB substrates. As only a few viral DUBs have been characterized thus far, we need to explore other viral DUBs that remain largely unknown. Moreover, chemical techniques may be employed to identify novel inhibitors of DUBs, and small-molecule antiviral drugs.

## Author Contributions

QZ, WG, and QJ wrote this manuscript. WZ revised this manuscript. All authors contributed to the article and approved the submitted version.

## Conflict of Interest

The authors declare that the research was conducted in the absence of any commercial or financial relationships that could be construed as a potential conflict of interest.

## Publisher’s Note

All claims expressed in this article are solely those of the authors and do not necessarily represent those of their affiliated organizations, or those of the publisher, the editors and the reviewers. Any product that may be evaluated in this article, or claim that may be made by its manufacturer, is not guaranteed or endorsed by the publisher.
